# 1-Methyl-4-[(*E*)-2-(3-hy­droxy-4-meth­oxy­phen­yl)ethen­yl]pyridinium 4-bromo­benzene­sulfonate monohydrate

**DOI:** 10.1107/S1600536813027244

**Published:** 2013-10-09

**Authors:** Suchada Chantrapromma, Pumsak Ruanwas, Boonwasana Jindawong, Hoong-Kun Fun

**Affiliations:** a Department of Chemistry, Faculty of Science, Prince of Songkla University, Hat-Yai, Songkhla 90112, Thailand; b X-ray Crystallography Unit, School of Physics, Universiti Sains Malaysia, 11800 USM, Penang, Malaysia

## Abstract

In the title hydrated salt, C_15_H_16_NO_2_^+^·C_6_H_4_BrO_3_S^−^·H_2_O, the cation exists in an *E* conformation with respect to the ethenyl bond and is almost planar, with a dihedral angle of 2.62 (12)° between the planes of the pyridinium and benzene rings. The meth­oxy substituent deviates slightly from the plane of its attached benzene ring [C_meth­yl_—O—C—C torsion angle = −11.6 (6)°]. In the crystal, the cations, anion and water mol­ecules are linked together into chains along [010] by O—H⋯O hydrogen bonds and weak C—H⋯O inter­actions. There is a short Br⋯O contact [3.029 (2) Å]. The crystal structure also features C—H⋯π inter­actions involving the benzene ring of the anion.

## Related literature

For bond-length data, see: Allen *et al.* (1987 ▶). For applications of stilbene derivatives, see: Belluti *et al.* (2010 ▶); Chanawanno *et al.* (2010 ▶); Frombaum *et al.* (2012 ▶); Hussain *et al.* (2009 ▶); Jindawong *et al.* (2005 ▶); Li *et al.* (2013 ▶); Ruanwas *et al.* (2010 ▶). For related structures, see: Chanawanno *et al.* (2009 ▶); Fun *et al.* (2011 ▶); Jindawong *et al.* (2005 ▶). For the stability of the temperature controller used in the data collection, see: Cosier & Glazer, (1986 ▶).
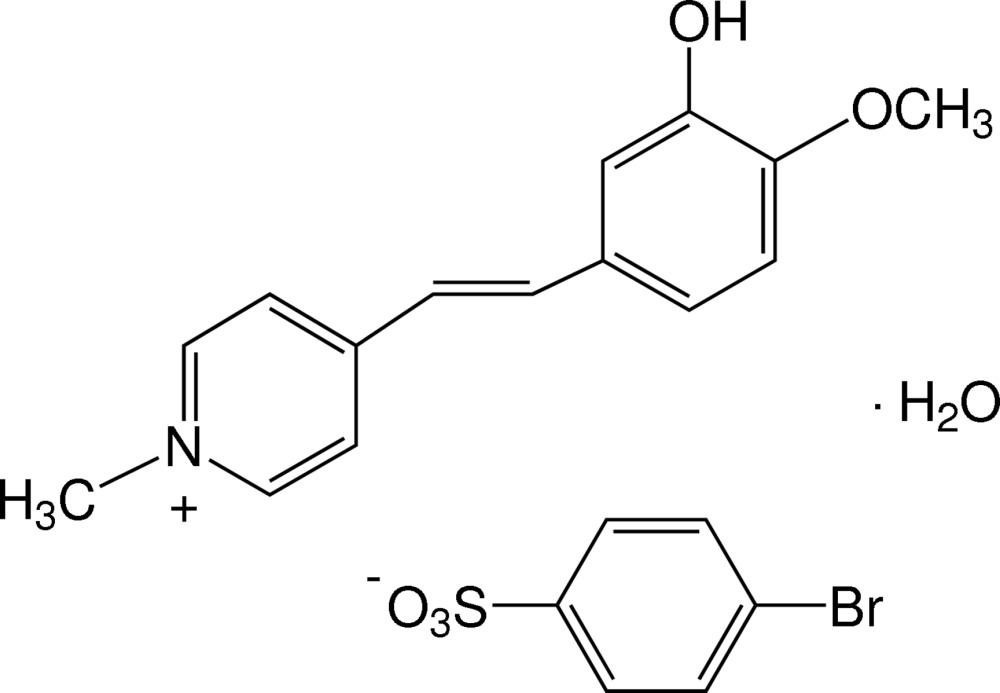


## Experimental

### 

#### Crystal data

C_15_H_16_NO_2_^+^·C_6_H_4_BrO_3_S^−^·H_2_O
*M* *_r_* = 496.37
Triclinic, 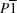

*a* = 9.7426 (7) Å
*b* = 9.8821 (7) Å
*c* = 11.8356 (8) Å
α = 80.107 (1)°
β = 73.140 (1)°
γ = 83.297 (1)°
*V* = 1071.60 (13) Å^3^
*Z* = 2
Mo *K*α radiation
μ = 2.05 mm^−1^
*T* = 100 K
0.54 × 0.51 × 0.16 mm

#### Data collection

Bruker APEXII CCD area-detector diffractometer
Absorption correction: multi-scan (*SADABS*; Bruker, 2005 ▶) *T*_min_ = 0.402, *T*_max_ = 0.728
5431 measured reflections
3717 independent reflections
3392 reflections with *I* > 2σ(*I*)
*R*_int_ = 0.027

#### Refinement

*R*[*F* ^2^ > 2σ(*F* ^2^)] = 0.043
*wR*(*F* ^2^) = 0.125
*S* = 1.05
3717 reflections
286 parameters
H atoms treated by a mixture of independent and constrained refinement
Δρ_max_ = 0.59 e Å^−3^
Δρ_min_ = −0.82 e Å^−3^

### 

Data collection: *APEX2* (Bruker, 2005 ▶); cell refinement: *APEX2*; data reduction: *SAINT* (Bruker, 2005 ▶); program(s) used to solve structure: *SHELXTL* (Sheldrick, 2008 ▶); program(s) used to refine structure: *SHELXTL*; molecular graphics: *SHELXTL*; software used to prepare material for publication: *SHELXTL*, *PLATON* (Spek, 2009 ▶) and *publCIF* (Westrip, 2010 ▶).

## Supplementary Material

Crystal structure: contains datablock(s) global, I. DOI: 10.1107/S1600536813027244/sj5354sup1.cif

Structure factors: contains datablock(s) I. DOI: 10.1107/S1600536813027244/sj5354Isup2.hkl

Supplementary material file. DOI: 10.1107/S1600536813027244/sj5354Isup3.cml

Additional supplementary materials:  crystallographic information; 3D view; checkCIF report

## Figures and Tables

**Table 1:** Hydrogen-bond geometry (Å, °) *Cg*_1_ is the centroid of the C1–C6 ring.

*D*—H⋯*A*	*D*—H	H⋯*A*	*D*⋯*A*	*D*—H⋯*A*
O1*W*—H1*W*1⋯O4^i^	0.80 (5)	2.52 (5)	3.015 (4)	122 (4)
O1*W*—H1*W*1⋯O5^i^	0.80 (5)	2.22 (5)	3.008 (4)	166 (4)
O1*W*—H2*W*1⋯O3	0.82 (5)	2.00 (5)	2.809 (4)	169 (5)
O4—H4*A*⋯O1*W* ^ii^	0.79 (4)	1.88 (4)	2.656 (4)	168 (3)
C2—H2*A*⋯O1^iii^	0.93	2.49	3.216 (3)	135
C5—H5*A*⋯O4	0.93	2.40	3.241 (3)	150
C18—H18*A*⋯O1^iv^	0.93	2.60	3.476 (5)	158
C10—H10*A*⋯*Cg*1^v^	0.93	2.78	3.672 (4)	160
C16—H16*A*⋯*Cg*1^vi^	0.93	2.72	3.543 (3)	148

Symmetry codes: (i) 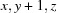; (ii) 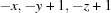; (iii) 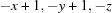; (iv) 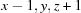; (v) 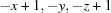; (vi) 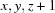.

**Fig. 1.**
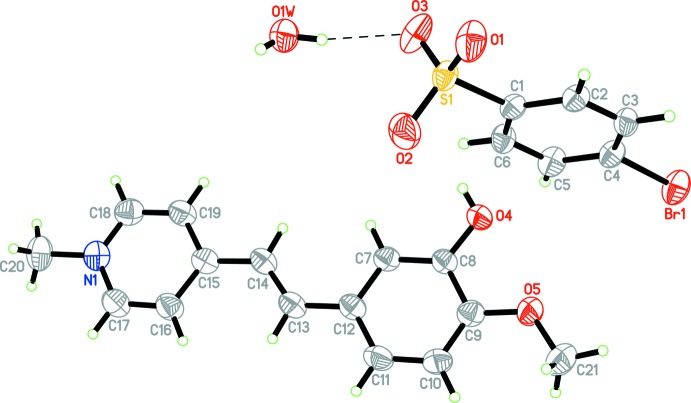
The molecular structure of the title compound, showing 40% probability displacement ellipsoids. A hydrogen bond is shown as a dashed line.

**Fig. 2.**
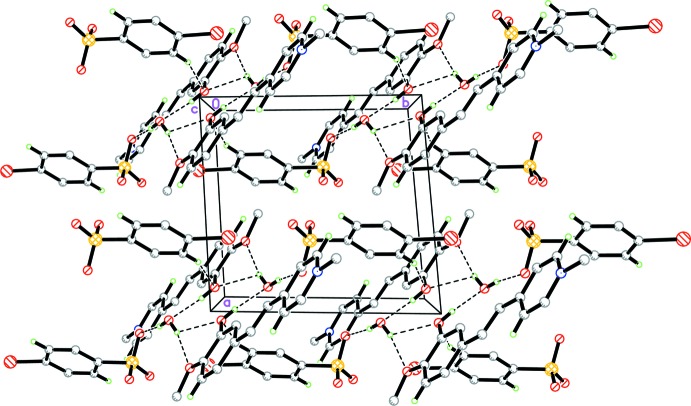
The crystal packing of the title compound viewed approximately along the c axis. Only H atoms involved in O—H···O hydrogen bonds and weak C—H···O interactions are shown for clarity. Hydrogen bonds are drawn as dashed lines.

## supplementary crystallographic information

### 1. Comment

The stilbene scaffold is a basic element for a number of biologically active natural and synthetic compounds. Stilbene-based compounds are extensively present in nature and have attracted chemists and biologists because of their wide range of biological activities, acting as antibacterial (Chanawanno *et al.*, 2010), anticancer (Belluti *et al.*, 2010) and antioxidant (Frombaum *et al.*, 2012) agents. In addition, some stilbenes also exhibit non-linear optical (Ruanwas *et al.*, 2010) and fluorescent properties (Li *et al.*, 2013). They are also generally used in the manufacturing industry as whitening agents (Hussain *et al.*, 2009). Due to these interesting properties, the title pyridinium-stilbene (I) was synthesized. We report herein the synthesis and crystal structure of (I).

The asymmetric unit of (I) consists of a C_15_H_16_NO_2_^+^ cation, a C_6_H_4_BrSO_3_^-^ anion and a H_2_O molecule (Fig. 1). All bond lengths (Allen *et al.*, 1987) and angles in both the cation and anion are normal and compare well with those found in closely related structures (Chanawanno *et al.*, 2009; Fun *et al.*, 2011; Jindawong *et al.*, 2005). The cation exists in an *E* configuration with respect to the C13 ═C14 double bond [1.326 (5) Å] and the C12—C13—C14—C15 torsion angle is 179.3 (3)°. The cation is essentially planar with a dihedral angle between the pyridinium and benzene rings of the cation of 2.62 (12)°. The hydroxy group lies close to the plane of the C7···C12 benzene ring whereas the methoxy group is slightly twisted from this plane with a C21–O5–C9–C10 torsion angle of -11.6 (6)°. The benzene ring of the 4-bromobenzenesulfonate anion makes dihedral angles of 80.35 (15) and 78.37 (15)° with pyridinium and benzene rings of the cation, respectively.

In the crystal packing (Fig. 2), intermolecular O—H···O hydrogen bonds form between the water molecule (O1W) and the O3 (sulfonate), O4 (hydroxy) and O5 (methoxy) atoms, and between the hydroxy substituent (O4) and the water molecule (O1W) (Table 1). Atoms O1 and O4 are involved in weak intermolecular C—H···O interactions (Table 1). The cations, anions and water molecules are linked into chains along [0 1 0] through these O–H···O and weak C—H···O hydrogen bonds (Fig. 2 and Table 1). The crystal structure is further stabilized by C—H···π interactions involving the centroid of the benzene ring of the cation (Table 1). A short Br···O contact [3.029 (2) Å; symmetry code +x, -1+y, z] also forms between adjacent anions.

### 2. Experimental

First of all, 1-methyl-4-[(*E*)-2-(3-hydroxy-4-methoxyphenyl)ethenyl]pyridinium iodide (compound A) was prepared by mixing a solution (1:1:1 molar ratio) of 1,4-dimethylpyridinium iodide (2.98 g, 12.68 mmol), isovanillin (1.96 g, 12.86 mmol) and piperidine (1.09 g, 12.80 mmol). The resulting solution was refluxed for 3 h under a nitrogen atmosphere. The solid which formed was filtered, washed with diethylether and recrystallized from methanol, to give brown crystals of compound A (3.99 g, 85% yield Mp. 503-504 K). Thereafter, the title compound was synthesized by mixing a solution of compound A (0.21 g, 0.58 mmol) in hot methanol (45 ml) and a solution of silver (I) 4-bromobenzenesulfonate (Jindawong *et al.*, 2005), (0.20 g, 0.58 mmol) in hot methanol (40 ml). The mixture yielded a yellow solid of silver iodide immediately. After stirring the mixture for 20 min, the precipitate of silver iodide was removed and the resulting yellow solution was evaporated to yield the title compound as a yellow solid (0.27 g, 94% yield). Yellow needle-shaped single crystals of the title compound suitable for *x*-ray structure determination were recrystalized from ethanol/methanol (1:1 *v*/*v*) by slow evaporation of the solvent at room temperature over several days, Mp. 511–512 K.

### 3. Refinement

Hydroxy and water H atoms were located in difference maps and refined isotropically. The remaining H atoms were positioned geometrically and allowed to ride on their parent atoms, with d(C—H) = 0.93 Å for aromatic and CH, and 0.96 Å for CH_3_ atoms. The *U*_iso_ values were constrained to be 1.5*U*_eq_ of the carrier atom for methyl H atoms and 1.2*U*_eq_ for the remaining H atoms. A rotating group model was used for the methyl groups.

### Crystal data

**Table:**

C_15_H_16_NO_2_^+^·C_6_H_4_BrO_3_S^−^·H_2_O	*Z* = 2
*M**_r_* = 496.37	*F*(000) = 508
Triclinic, *P*1	*D*_x_ = 1.538 Mg m^−^^3^
Hall symbol: -P 1	Melting point = 511–512 K
*a* = 9.7426 (7) Å	Mo *K*α radiation, λ = 0.71073 Å
*b* = 9.8821 (7) Å	Cell parameters from 3717 reflections
*c* = 11.8356 (8) Å	θ = 2.1–25.0°
α = 80.107 (1)°	µ = 2.05 mm^−^^1^
β = 73.140 (1)°	*T* = 100 K
γ = 83.297 (1)°	Needle, yellow
*V* = 1071.60 (13) Å^3^	0.54 × 0.51 × 0.16 mm

### Data collection

**Table:**

Bruker APEXII CCD area-detector diffractometer	3717 independent reflections
Radiation source: sealed tube	3392 reflections with *I* > 2σ(*I*)
Graphite monochromator	*R*_int_ = 0.027
φ and ω scans	θ_max_ = 25.0°, θ_min_ = 2.1°
Absorption correction: multi-scan (*SADABS*; Bruker, 2005)	*h* = −11→11
*T*_min_ = 0.402, *T*_max_ = 0.728	*k* = −11→11
5431 measured reflections	*l* = −14→12

### Refinement

**Table:**

Refinement on *F*^2^	Secondary atom site location: difference Fourier map
Least-squares matrix: full	Hydrogen site location: inferred from neighbouring sites
*R*[*F*^2^ > 2σ(*F*^2^)] = 0.043	H atoms treated by a mixture of independent and constrained refinement
*wR*(*F*^2^) = 0.125	*w* = 1/[σ^2^(*F*_o_^2^) + (0.0865*P*)^2^ + 0.2408*P*] where *P* = (*F*_o_^2^ + 2*F*_c_^2^)/3
*S* = 1.05	(Δ/σ)_max_ = 0.001
3717 reflections	Δρ_max_ = 0.59 e Å^−^^3^
286 parameters	Δρ_min_ = −0.82 e Å^−^^3^
0 restraints	Extinction correction: *SHELXTL* (Sheldrick, 2008), Fc^*^=kFc[1+0.001xFc^2^λ^3^/sin(2θ)]^-1/4^
Primary atom site location: structure-invariant direct methods	Extinction coefficient: 0.026 (3)

### Special details

**Table:**

Experimental. The crystal was placed in the cold stream of an Oxford Cryosystems Cobra open-flow nitrogen cryostat (Cosier & Glazer, 1986) operating at 100.0 (1) K.
Geometry. All esds (except the esd in the dihedral angle between two l.s. planes) are estimated using the full covariance matrix. The cell esds are taken into account individually in the estimation of esds in distances, angles and torsion angles; correlations between esds in cell parameters are only used when they are defined by crystal symmetry. An approximate (isotropic) treatment of cell esds is used for estimating esds involving l.s. planes.
Refinement. Refinement of F^2^ against ALL reflections. The weighted R-factor wR and goodness of fit S are based on F^2^, conventional R-factors R are based on F, with F set to zero for negative F^2^. The threshold expression of F^2^ > 2sigma(F^2^) is used only for calculating R-factors(gt) etc. and is not relevant to the choice of reflections for refinement. R-factors based on F^2^ are statistically about twice as large as those based on F, and R- factors based on ALL data will be even larger.

### Fractional atomic coordinates and isotropic or equivalent isotropic displacement parameters (Å^2^)

**Table:**

	*x*	*y*	*z*	*U*_iso_*/*U*_eq_	
Br1	0.33252 (3)	−0.08552 (3)	0.17801 (3)	0.05706 (18)	
S1	0.31851 (7)	0.54351 (6)	0.25594 (6)	0.0432 (2)	
O1	0.3962 (3)	0.6148 (2)	0.14279 (19)	0.0682 (7)	
O2	0.3892 (3)	0.5389 (2)	0.3481 (2)	0.0690 (6)	
O3	0.1683 (2)	0.5911 (2)	0.2912 (3)	0.0717 (7)	
O4	0.0748 (2)	0.0170 (3)	0.57781 (19)	0.0612 (6)	
H4A	0.021 (4)	0.083 (4)	0.573 (3)	0.060 (10)*	
O5	0.3044 (3)	−0.1435 (3)	0.5684 (2)	0.0673 (7)	
N1	−0.1966 (3)	0.4820 (3)	1.2500 (2)	0.0552 (6)	
C1	0.3205 (3)	0.3703 (3)	0.2328 (2)	0.0370 (5)	
C2	0.3903 (3)	0.3303 (3)	0.1220 (2)	0.0397 (5)	
H2A	0.4362	0.3947	0.0595	0.048*	
C3	0.3917 (3)	0.1959 (3)	0.1045 (2)	0.0413 (6)	
H3A	0.4375	0.1692	0.0303	0.050*	
C4	0.3239 (3)	0.1009 (3)	0.1991 (2)	0.0414 (6)	
C5	0.2534 (3)	0.1391 (3)	0.3101 (2)	0.0473 (6)	
H5A	0.2081	0.0745	0.3728	0.057*	
C6	0.2516 (3)	0.2742 (3)	0.3261 (2)	0.0434 (6)	
H6A	0.2040	0.3013	0.3999	0.052*	
C7	0.0825 (3)	0.0955 (3)	0.7566 (2)	0.0459 (6)	
H7A	−0.0007	0.1528	0.7583	0.055*	
C8	0.1366 (3)	0.0180 (3)	0.6658 (2)	0.0450 (6)	
C9	0.2618 (3)	−0.0698 (3)	0.6621 (3)	0.0512 (7)	
C10	0.3307 (3)	−0.0750 (4)	0.7487 (3)	0.0607 (8)	
H10A	0.4147	−0.1313	0.7460	0.073*	
C11	0.2752 (4)	0.0035 (4)	0.8403 (3)	0.0630 (9)	
H11A	0.3226	−0.0016	0.8988	0.076*	
C12	0.1506 (3)	0.0898 (3)	0.8470 (2)	0.0483 (7)	
C13	0.0935 (3)	0.1690 (3)	0.9448 (3)	0.0536 (7)	
H13A	0.1457	0.1596	1.0006	0.064*	
C14	−0.0245 (3)	0.2534 (3)	0.9641 (3)	0.0518 (7)	
H14A	−0.0767	0.2641	0.9082	0.062*	
C15	−0.0802 (3)	0.3305 (3)	1.0634 (2)	0.0482 (6)	
C16	−0.0137 (3)	0.3290 (4)	1.1535 (3)	0.0601 (8)	
H16A	0.0721	0.2758	1.1515	0.072*	
C17	−0.0727 (4)	0.4041 (4)	1.2442 (3)	0.0621 (8)	
H17A	−0.0265	0.4016	1.3032	0.074*	
C18	−0.2637 (4)	0.4864 (4)	1.1655 (3)	0.0610 (8)	
H18A	−0.3492	0.5408	1.1698	0.073*	
C19	−0.2093 (3)	0.4127 (4)	1.0733 (3)	0.0580 (8)	
H19A	−0.2585	0.4169	1.0160	0.070*	
C20	−0.2569 (5)	0.5637 (4)	1.3496 (3)	0.0757 (10)	
H20A	−0.3572	0.5877	1.3571	0.114*	
H20B	−0.2069	0.6460	1.3336	0.114*	
H20C	−0.2456	0.5101	1.4225	0.114*	
C21	0.4426 (4)	−0.2138 (5)	0.5445 (4)	0.0830 (12)	
H21A	0.4610	−0.2535	0.4725	0.125*	
H21B	0.4467	−0.2854	0.6097	0.125*	
H21C	0.5137	−0.1501	0.5350	0.125*	
O1W	0.0853 (3)	0.7571 (3)	0.4734 (2)	0.0615 (6)	
H1W1	0.143 (5)	0.796 (5)	0.490 (4)	0.087 (16)*	
H2W1	0.120 (5)	0.706 (5)	0.423 (4)	0.082 (13)*	

### Atomic displacement parameters (Å^2^)

**Table:**

	*U*^11^	*U*^22^	*U*^33^	*U*^12^	*U*^13^	*U*^23^
Br1	0.0620 (3)	0.0341 (2)	0.0688 (3)	−0.01219 (14)	−0.00293 (16)	−0.01084 (14)
S1	0.0501 (4)	0.0321 (4)	0.0444 (4)	−0.0052 (3)	−0.0061 (3)	−0.0074 (3)
O1	0.1028 (18)	0.0350 (11)	0.0525 (12)	−0.0186 (11)	0.0039 (11)	−0.0027 (9)
O2	0.0927 (18)	0.0552 (13)	0.0709 (14)	−0.0150 (12)	−0.0346 (13)	−0.0139 (11)
O3	0.0546 (14)	0.0496 (13)	0.1069 (18)	0.0051 (11)	−0.0082 (12)	−0.0308 (12)
O4	0.0587 (13)	0.0770 (16)	0.0564 (13)	0.0155 (12)	−0.0285 (10)	−0.0243 (11)
O5	0.0547 (13)	0.0828 (17)	0.0703 (14)	0.0182 (12)	−0.0225 (11)	−0.0339 (12)
N1	0.0515 (14)	0.0595 (16)	0.0491 (13)	−0.0117 (12)	−0.0023 (11)	−0.0081 (11)
C1	0.0358 (12)	0.0314 (12)	0.0415 (13)	−0.0042 (9)	−0.0072 (10)	−0.0035 (10)
C2	0.0385 (13)	0.0331 (13)	0.0404 (13)	−0.0050 (10)	−0.0029 (10)	0.0011 (10)
C3	0.0414 (13)	0.0413 (14)	0.0374 (12)	−0.0029 (11)	−0.0031 (10)	−0.0088 (10)
C4	0.0395 (13)	0.0308 (12)	0.0504 (14)	−0.0062 (10)	−0.0066 (11)	−0.0044 (10)
C5	0.0489 (15)	0.0361 (14)	0.0483 (15)	−0.0130 (11)	−0.0004 (12)	0.0010 (11)
C6	0.0436 (14)	0.0439 (14)	0.0362 (12)	−0.0063 (11)	0.0008 (10)	−0.0065 (11)
C7	0.0360 (13)	0.0552 (16)	0.0451 (14)	−0.0043 (11)	−0.0100 (11)	−0.0046 (12)
C8	0.0405 (14)	0.0519 (16)	0.0424 (14)	−0.0059 (11)	−0.0114 (11)	−0.0049 (11)
C9	0.0434 (15)	0.0558 (17)	0.0542 (16)	−0.0043 (13)	−0.0131 (12)	−0.0074 (13)
C10	0.0456 (16)	0.073 (2)	0.067 (2)	0.0074 (15)	−0.0225 (15)	−0.0151 (16)
C11	0.0545 (18)	0.082 (2)	0.0612 (19)	0.0042 (16)	−0.0302 (15)	−0.0147 (17)
C12	0.0418 (14)	0.0584 (18)	0.0457 (14)	−0.0108 (13)	−0.0127 (11)	−0.0045 (12)
C13	0.0492 (16)	0.069 (2)	0.0475 (15)	−0.0099 (14)	−0.0185 (13)	−0.0073 (14)
C14	0.0492 (16)	0.0628 (19)	0.0464 (15)	−0.0109 (14)	−0.0160 (12)	−0.0062 (13)
C15	0.0410 (14)	0.0563 (17)	0.0451 (14)	−0.0122 (12)	−0.0089 (11)	−0.0003 (13)
C16	0.0465 (16)	0.081 (2)	0.0544 (17)	0.0015 (15)	−0.0150 (13)	−0.0177 (16)
C17	0.0539 (18)	0.081 (2)	0.0534 (17)	−0.0055 (16)	−0.0158 (14)	−0.0145 (16)
C18	0.0500 (17)	0.067 (2)	0.0607 (18)	−0.0004 (15)	−0.0111 (14)	−0.0061 (15)
C19	0.0522 (17)	0.071 (2)	0.0527 (17)	−0.0032 (15)	−0.0192 (14)	−0.0075 (15)
C20	0.080 (2)	0.079 (3)	0.064 (2)	−0.005 (2)	−0.0029 (18)	−0.0287 (19)
C21	0.064 (2)	0.090 (3)	0.095 (3)	0.023 (2)	−0.022 (2)	−0.036 (2)
O1W	0.0623 (14)	0.0660 (15)	0.0545 (13)	0.0033 (12)	−0.0109 (11)	−0.0183 (11)

### Geometric parameters (Å, º)

**Table:**

Br1—C4	1.890 (3)	C9—C10	1.370 (4)
S1—O1	1.440 (2)	C10—C11	1.386 (5)
S1—O2	1.441 (2)	C10—H10A	0.9300
S1—O3	1.445 (2)	C11—C12	1.390 (4)
S1—C1	1.777 (2)	C11—H11A	0.9300
O4—C8	1.348 (3)	C12—C13	1.448 (4)
O4—H4A	0.79 (4)	C13—C14	1.326 (5)
O5—C9	1.369 (4)	C13—H13A	0.9300
O5—C21	1.416 (4)	C14—C15	1.449 (4)
N1—C18	1.338 (4)	C14—H14A	0.9300
N1—C17	1.344 (4)	C15—C16	1.397 (4)
N1—C20	1.483 (4)	C15—C19	1.402 (4)
C1—C6	1.390 (4)	C16—C17	1.361 (5)
C1—C2	1.391 (4)	C16—H16A	0.9300
C2—C3	1.377 (4)	C17—H17A	0.9300
C2—H2A	0.9300	C18—C19	1.359 (5)
C3—C4	1.388 (4)	C18—H18A	0.9300
C3—H3A	0.9300	C19—H19A	0.9300
C4—C5	1.389 (4)	C20—H20A	0.9600
C5—C6	1.377 (4)	C20—H20B	0.9600
C5—H5A	0.9300	C20—H20C	0.9600
C6—H6A	0.9300	C21—H21A	0.9600
C7—C8	1.371 (4)	C21—H21B	0.9600
C7—C12	1.404 (4)	C21—H21C	0.9600
C7—H7A	0.9300	O1W—H1W1	0.80 (5)
C8—C9	1.406 (4)	O1W—H2W1	0.81 (4)
			
O1—S1—O2	112.81 (16)	C10—C11—C12	121.9 (3)
O1—S1—O3	112.92 (16)	C10—C11—H11A	119.1
O2—S1—O3	113.31 (16)	C12—C11—H11A	119.1
O1—S1—C1	105.43 (12)	C11—C12—C7	117.4 (3)
O2—S1—C1	106.15 (12)	C11—C12—C13	120.8 (3)
O3—S1—C1	105.34 (12)	C7—C12—C13	121.8 (3)
C8—O4—H4A	111 (3)	C14—C13—C12	127.6 (3)
C9—O5—C21	118.7 (3)	C14—C13—H13A	116.2
C18—N1—C17	119.8 (3)	C12—C13—H13A	116.2
C18—N1—C20	120.5 (3)	C13—C14—C15	126.5 (3)
C17—N1—C20	119.6 (3)	C13—C14—H14A	116.7
C6—C1—C2	119.9 (2)	C15—C14—H14A	116.7
C6—C1—S1	119.64 (19)	C16—C15—C19	116.0 (3)
C2—C1—S1	120.50 (19)	C16—C15—C14	124.2 (3)
C3—C2—C1	120.3 (2)	C19—C15—C14	119.8 (3)
C3—C2—H2A	119.8	C17—C16—C15	120.9 (3)
C1—C2—H2A	119.8	C17—C16—H16A	119.5
C2—C3—C4	119.1 (2)	C15—C16—H16A	119.5
C2—C3—H3A	120.4	N1—C17—C16	121.1 (3)
C4—C3—H3A	120.4	N1—C17—H17A	119.4
C3—C4—C5	121.3 (2)	C16—C17—H17A	119.4
C3—C4—Br1	119.4 (2)	N1—C18—C19	121.2 (3)
C5—C4—Br1	119.21 (19)	N1—C18—H18A	119.4
C6—C5—C4	119.0 (2)	C19—C18—H18A	119.4
C6—C5—H5A	120.5	C18—C19—C15	121.0 (3)
C4—C5—H5A	120.5	C18—C19—H19A	119.5
C5—C6—C1	120.4 (2)	C15—C19—H19A	119.5
C5—C6—H6A	119.8	N1—C20—H20A	109.5
C1—C6—H6A	119.8	N1—C20—H20B	109.5
C8—C7—C12	121.1 (3)	H20A—C20—H20B	109.5
C8—C7—H7A	119.5	N1—C20—H20C	109.5
C12—C7—H7A	119.5	H20A—C20—H20C	109.5
O4—C8—C7	123.9 (2)	H20B—C20—H20C	109.5
O4—C8—C9	115.8 (2)	O5—C21—H21A	109.5
C7—C8—C9	120.3 (2)	O5—C21—H21B	109.5
O5—C9—C10	125.6 (3)	H21A—C21—H21B	109.5
O5—C9—C8	115.0 (2)	O5—C21—H21C	109.5
C10—C9—C8	119.4 (3)	H21A—C21—H21C	109.5
C9—C10—C11	120.0 (3)	H21B—C21—H21C	109.5
C9—C10—H10A	120.0	H1W1—O1W—H2W1	115 (4)
C11—C10—H10A	120.0		
			
O1—S1—C1—C6	−179.1 (2)	C7—C8—C9—C10	−1.3 (5)
O2—S1—C1—C6	61.0 (2)	O5—C9—C10—C11	−179.0 (3)
O3—S1—C1—C6	−59.5 (2)	C8—C9—C10—C11	1.4 (5)
O1—S1—C1—C2	0.9 (3)	C9—C10—C11—C12	−0.5 (6)
O2—S1—C1—C2	−119.0 (2)	C10—C11—C12—C7	−0.4 (5)
O3—S1—C1—C2	120.5 (2)	C10—C11—C12—C13	178.9 (3)
C6—C1—C2—C3	−0.1 (4)	C8—C7—C12—C11	0.5 (4)
S1—C1—C2—C3	179.9 (2)	C8—C7—C12—C13	−178.8 (3)
C1—C2—C3—C4	−0.6 (4)	C11—C12—C13—C14	−178.9 (3)
C2—C3—C4—C5	0.8 (4)	C7—C12—C13—C14	0.4 (5)
C2—C3—C4—Br1	−177.19 (19)	C12—C13—C14—C15	179.3 (3)
C3—C4—C5—C6	−0.2 (4)	C13—C14—C15—C16	1.6 (5)
Br1—C4—C5—C6	177.8 (2)	C13—C14—C15—C19	−178.1 (3)
C4—C5—C6—C1	−0.6 (4)	C19—C15—C16—C17	−0.3 (5)
C2—C1—C6—C5	0.8 (4)	C14—C15—C16—C17	−179.9 (3)
S1—C1—C6—C5	−179.3 (2)	C18—N1—C17—C16	0.0 (5)
C12—C7—C8—O4	178.7 (3)	C20—N1—C17—C16	−179.4 (4)
C12—C7—C8—C9	0.4 (5)	C15—C16—C17—N1	0.1 (6)
C21—O5—C9—C10	−11.6 (6)	C17—N1—C18—C19	0.3 (5)
C21—O5—C9—C8	168.1 (3)	C20—N1—C18—C19	179.6 (3)
O4—C8—C9—O5	0.6 (4)	N1—C18—C19—C15	−0.5 (6)
C7—C8—C9—O5	179.0 (3)	C16—C15—C19—C18	0.5 (5)
O4—C8—C9—C10	−179.8 (3)	C14—C15—C19—C18	−179.8 (3)

### Hydrogen-bond geometry (Å, º)

*Cg*_1_ is the centroid of the C1–C6 ring.

**Table:**

*D*—H···*A*	*D*—H	H···*A*	*D*···*A*	*D*—H···*A*
O1*W*—H1*W*1···O4^i^	0.80 (5)	2.52 (5)	3.015 (4)	122 (4)
O1*W*—H1*W*1···O5^i^	0.80 (5)	2.22 (5)	3.008 (4)	166 (4)
O1*W*—H2*W*1···O3	0.82 (5)	2.00 (5)	2.809 (4)	169 (5)
O4—H4*A*···O1*W*^ii^	0.79 (4)	1.88 (4)	2.656 (4)	168 (3)
C2—H2*A*···O1^iii^	0.93	2.49	3.216 (3)	135
C5—H5*A*···O4	0.93	2.40	3.241 (3)	150
C18—H18*A*···O1^iv^	0.93	2.60	3.476 (5)	158
C10—H10*A*···*Cg*1^v^	0.93	2.78	3.672 (4)	160
C16—H16*A*···*Cg*1^vi^	0.93	2.72	3.543 (3)	148

Symmetry codes: (i) *x*, *y*+1, *z*; (ii) −*x*, −*y*+1, −*z*+1; (iii) −*x*+1, −*y*+1, −*z*; (iv) *x*−1, *y*, *z*+1; (v) −*x*+1, −*y*, −*z*+1; (vi) *x*, *y*, *z*+1.
